# Eosinophilic enterocolitis in duodenum, ileum, and colon: A case report

**DOI:** 10.1016/j.heliyon.2024.e26885

**Published:** 2024-03-02

**Authors:** Isabella Van-Londoño, Camilo Ramírez-Giraldo, Julio César Martínez Echeverri, Juan José Villany-Sarmiento, Laura Marcela Fino-Velásquez

**Affiliations:** a Universidad del Rosario, Bogotá, Colombia; b Hospital Universitario Mayor – Méderi, Bogotá, Colombia

**Keywords:** Eosinophilic gastroenteritis, Eosinophilic enterocolitis, Hypereosinophilic syndrome, Gastroenterology

## Abstract

Eosinophilic gastroenteritis (EGE) is a rare disease which mainly consists of an abnormal eosinophile infiltration of the gastrointestinal tract. It’s classified according to its location: eosinophilic esophagitis, eosinophilic gastritis, eosinophilic enteritis (including duodenum, jejunum and/or ileum) and eosinophilic colitis and degree of infiltration (mucosal, muscular, serosal). Depending on eosinophile concentration, type of EGE and the patient’s condition it may manifest with different clinical presentations such as functional dyspepsia, abdominal pain, irritability, hypoproteinemia, diarrhea, anemia, among others. Few research has been done on such an uncommon pathology to the extent that treatment evidence is mostly limited to small case series. This case study reports an infrequent presentation of EGE in the small and large intestine as an undifferentiated gastrointestinal disease and successful corticoid management given to the patient in order to further broaden knowledge on this subject and facilitate an established clinical conduct for the treating physician.

## 1 Introduction

Eosinophilic gastroenteritis (EGE) is a rare infiltrative disorder characterized by an increase in eosinophil numbers and is subclassified by the regions of the gastrointestinal (GI) tract it infilters into eosinophilic esophagitis, gastritis, enteritis, and colitis. These cells cause an invasion of the different layers that are part of the gastrointestinal tract. Epidemiology on EGE is still uncertain, and although hundreds of cases and small case series have been reported worldwide, reported data is contradictory, with some studies reporting a higher male:female ratio, most commonly between 30 and 40 years of age. Incidence also varies depending on study origin and sample, suggesting that results may be underpowered by the relatively small number of patients with the disease [[1], [2]]. The most common symptoms in adults include diarrhea, nausea, abdominal pain and vomiting but can also include obstructive gastrointestinal disease, granuloma, or visceral perforation in worst case scenarios due to eosinophil’s inflammatory properties. The pathophysiology and the clinical presentation of this disease both remain unclear. However, the literature has been able to highlight the multiple roles of Ig-E responses in the gastrointestinal system and thus have an idea on possible complications [5].

Multiple differential diagnoses can cause an eosinophilic infiltration of the GI tract, out of which they are usually of infectious, autoimmune, and neoplastic origin [4,6,7]. As a result, all these different diseases must be considered and discarded before diagnosing EGE. Current literature is mostly limited to case reports however there isn’t any research underlining a treatment scheme to be followed (due to the absence of controlled trials) and therapies shown on these studies seem to be mostly experimental and reigned by expert’s criteria [4]. Here, we present the case of a 32-year-old female patient coursing with eosinophilic enterocolitis in duodenum, ileum and colon presenting as an undifferentiated gastrointestinal disease without a previous history of allergies, it is an unusual and exceedingly rare case considering the areas infiltrated in the gastrointestinal tract, the extent of its infiltration and patient medical history.

## 2 Case presentation

**Fig. 1:**
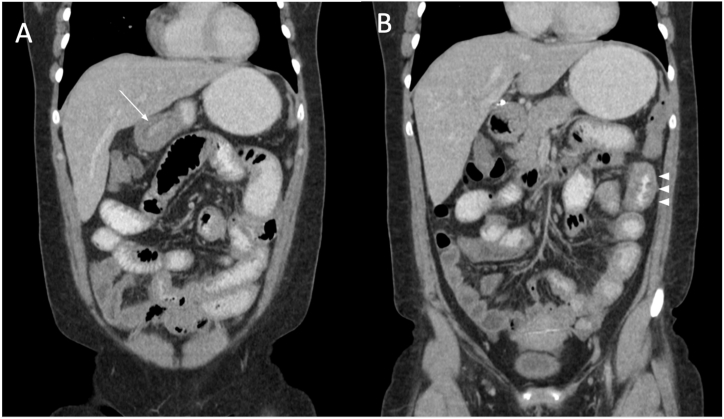
**“Abdominal CT scan, coronal plane”.** The main findings in the abdominal CT were segmental, concentric and multifocal thickening of small bowel, **A.** with greater involvement of the pylorus (white arrow) and **B**., proximal jejunum (arrowheads), causing a slight obstructive effect. A small amount of ascitic fluid was found in the pelvis, not susceptible for drainage.

We present the case of a 32-year-old female patient of Hispanic origin with a history of prehypertension, chronic gastritis and past treatment for H. pylori, without previous history of allergies or relevant family history, admitted to the emergency room due to 20-day symptomatology of pressurizing cholic-like abdominal pain in right flank and hypogastrium associated to multiple emetic episodes and diarrheic stools previously treated at a different health facility with Metronidazole and Cefalexin suspecting parasitic infection with no symptom relief. She was admitted to our institution and initial laboratory tests included a negative chorionic gonadotropin beta-subunit (bHCG), liver function tests with slightly elevated alkaline phosphatase and transaminases, normal renal function tests, and a blood test reporting leukocytosis due to an exceedingly high eosinophile count (6370 cells/mcL). Due to pain location and suspicion of a possibly surgical-related acute abdomen an abdominal computed tomography (CT) scan was taken (Fig. 1 [A-B]).

Patient was given analgesics and antisecretory medicine, nonetheless the patient persisted with symptoms including low oral tolerance, abdominal pain, and diarrheic stools. A coprological stool test, HIV serology and gastrointestinal film array were taken considering possible infectious jejunitis, all of them reporting negative results.

**Fig. 2:**
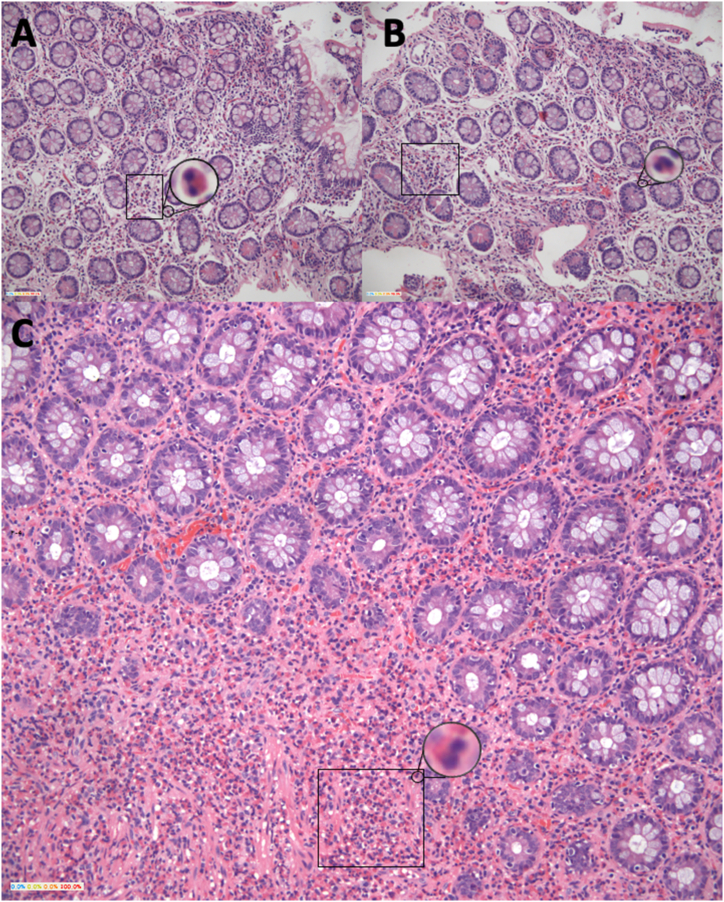
**“Intestinal biopsies”. A, B. Small intestine. C. Large intestine.** Intestinal mucosa with a count of >70 eosinophils (rectangle, amplified) per high power field invading mucosa and submucosa with lymphoid aggregates formation. No microorganism, dysplasia or malignancy can be observed. Hematoxylin-eosin stain.

The patient was nevertheless given antimicrobial treatment with a 3-day albendazole and a 5-day ampicillin/sulbactam scheme and was given a strict diet devoid of gluten and dairy suspecting inflammatory bowel disease versus infection. Peripheric blood smear indicated no alteration of cellular lines, thorax radiography reported no abnormalities and fecal calprotectin was reported as elevated. Patient’s laboratory tests controls remained stable, but she persisted with diarrheic stools and itching sensation in lower extremities, as a result metronidazole and antihistaminic therapy were administered. Additional upper gastrointestinal tract endoscopy and colonoscopy were performed to further explore gastrointestinal tract inflammation. The esophagogastroduodenoscopy showed marked thickening of duodenal mucosa narrowing its gauge and erythematous folds with superficial erosive lesions. A colonoscopy showed mild erythematous patches in distal ileum, and a patch in both sigmoid colon and rectum of edematous mucosa and marked erythema with loss of mucosa transparency. Biopsies of duodenum, ileum and colon were taken. Abdominal pain and nausea associated with food intake persisted. The biopsies' histology results evidenced eosinophilic mucosal and submucosal infiltration in duodenum, ileum, and colon of over 70 eosinophils per camp high power field (Fig. 2 [A-C]) while the stomach biopsy showed no alterations.

Due to both colonoscopy and endoscopy findings indicating possible rheumatological disease, loperamide was suspended and P-ANCA and C-ANCA antibodies were solicited to explore possible vasculitis but were reported as negative. Daily blood tests persisted with fluctuating eosinophile levels between 5000 and 8000 cells per microliter (cells/mcL). Due to biopsy results, patient’s symptoms, and negative antibody tests EGE diagnosis was suspected.

**Fig. 3:**
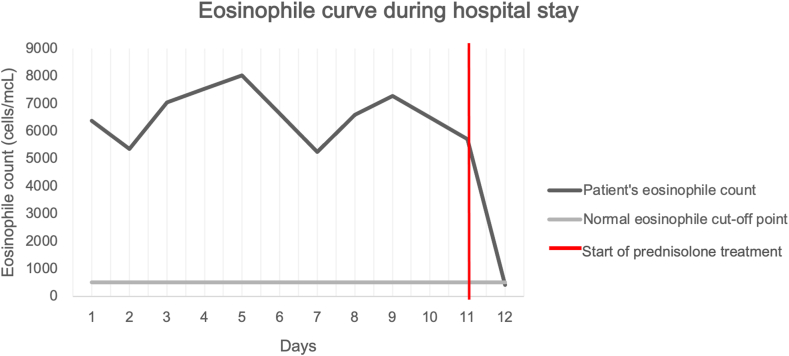
Patient's eosinophilic curve during hospital stay.

Treatment with low-dose corticoids (Prednisolone 10mg orally) was administered after previous deworming with ivermectin to discard possible strongyloidiasis. Symptoms improved almost immediately and eosinophile peripheric levels dropped in the following 24 hours after corticoid therapy was administered, the patient’s eosinophilic curve during hospital stay can be seen in (Fig. 3). She was given prompt hospital discharge. The patient was satisfied with both symptom relief and given treatment and referred only mild abdominal pain after a five-month telephonic follow-up when the initial 30-day corticoid treatment was suspended and is currently awaiting tests for an enteroscopy, IgE serum levels and a FIP1L1-PDGFRA test.

## 3 Discussion

EGE refers to a hyper eosinophilic infiltration of any part of the gastrointestinal tract, most commonly occurring in esophagus, stomach, and duodenum [8]. Due to it being a poorly understood disease with no clear pathophysiology or risk factors established to present date, it is considered a diagnosis of exclusion based on three criteria: suspicious clinical symptoms, exclusion of other pathologies with similar findings and histological evidence of eosinophilic infiltration of the gastrointestinal tract [9]. On the latter there is not a standard cut-off point for an abnormal mucosal eosinophil count, however a value over 20–30 eosinophils per field has been proven highly specific in some of the literature [[9], [10], [11]].

Eosinophils are normally present in the gastrointestinal tract’s mucosa and serve various roles in immunity and inflammation. Around 70% of EGE cases have been associated to a history of allergies (food or drug allergies, asthma, eczema, rhinitis, atopy, elevated IgE serum levels) and as a result, it has been proposed as a possible trigger for EGE, suggesting that T-cell imbalance due to hypersensitivity may cause an increase in cytokine production and may be a possible cause of increased IgE synthesis and thus subsequent eosinophilia [5,12]. These patients tend to have peripheral eosinophilia, T cell proliferation in the lamina propria, secretion of IL-13 in response to milk protein, abundant eosinophiles in the gastrointestinal tract and a dramatic response to steroids [8,12,13].

In nearly 50% of patients diarrhea, nausea, abdominal pain, and vomiting are present with some overlap [12], however they can also include obstructive gastrointestinal disease, ascites, granuloma, or visceral perforation in worst case scenarios due to eosinophil’s inflammatory properties [3,14,15], highlighting the urgency of effective therapy to prevent complications. Around 300 cases have been documented since its initial description by Kaijser in 1937 [16], out of which small intestine and colon remain the rarest and least reported.

Diagnostic images are essential in the approach to eosinophilic enteritis, since they allow us to rule out multiple differential diagnoses. Imaging findings in EGE are nonspecific, but include thickening of the bowel walls, stenotic areas, lymphadenitis, mesenteritis, and ascites depending on the chronicity of the disease and the layer involved (submucusosa, mucosa, serosa) [17].

In our case, EGE compromise was distributed in patches throughout the lower gastrointestinal tract (duodenum, distal ileum, and sigmoid colon) in mucosa and submucosa. Jejunum compromise was suspected because of tomography report however due to the impossibility of taking an endoscopic jejunum biopsy, eosinophilic compromise could not be confirmed. This case is unique due to the patient not having a history of allergies and the rare eosinophilic infiltrative presentation in the intestinal mucosa, respecting most common areas of involvement (esophagus, stomach), even more so considering patient’s previous history of chronic gastritis and H. pylori infection, which would suggest upper rather than lower gastrointestinal compromise [13]. The patient’s symptoms such as watery stools and vomiting can be attributed to small intestine compromise and degree of eosinophil infiltration, however pain localization does not correlate to the diffuse eosinophilic infiltration of the intestine, while the small amount of ascitic fluid in patient’s right fossa may indicate progression into the serosal layer, nonetheless treatment was given before these symptoms could advance any further [18].

Multiple diseases can present with eosinophilia in the intestinal mucosa and as a result were considered before EGE diagnosis. Rheumatological conditions such as hyper eosinophilic syndrome, systemic vasculitis, polymyositis, and Churg-Strauss syndrome were ruled out due to laboratory and clinical findings out of which we highlight the lack of systemic compromise, ANCA negative results, lack of general muscle weakening and lack of respiratory system compromise, respectively [19,20]. Inflammatory bowel diseases may also present with gastrointestinal symptoms and eosinophilic mucosal infiltration however due to negative antibody tests, form of presentation, no possible food trigger, no disease chronicity and no typical signs of the disease (food malabsorption, bloody stools, malnutrition, etc.) these were excluded as well [[21], [22], [23]].

Infectious causes are another well-known cause of both serologic and intestinal eosinophilic infiltration but were also discarded due to the gastrointestinal film array [19], and both the stool test and biopsies had no findings of any microorganisms or ova. Strongyloidiasis is another important differential diagnosis that must be considered but diagnosis is difficult as serial coprological tests are needed to evidence its growth, thus patient was dewormed regardless of the absence of a positive test [6].

Steroid therapy has long been the first option treatment when faced with EGE, with studies yielding positive results in studies on patients with eosinophilic esophagitis [24]. In our case, patient’s clinical response was almost immediate with absolute symptom relief after starting a low-dose corticoid treatment [4]. Even though a definite etiology has not been determined and patient’s history didn’t indicate towards any possible trigger, patient’s response to treatment may support the IgE hypersensitivity theory, highlighting the importance of considering EGE diagnosis in any patients consulting with gastrointestinal symptoms and peripheral eosinophilia, regardless of previous medical history or ambiguous symptomatology.

Limitations for this case report include the impossibility to obtain a jejunum biopsy in our institution as we lack the resources for an enteroscopy, lack of IgE serum levels and strongyloidiasis testing, and the lack of any long-term follow-up data on the patient. The CARE Checklist has been completed by the authors for this case report, attached as supplementary material.

## Funding

This research did not receive any specific grant from funding agencies in the public, commercial, or nonprofit sectors.

## Statement of ethics

Ethical compliance with the World Medical Association Declaration of Helsinki, current legislation on research Res. 008430-1993 and Res. 2378-2008 (Colombia) and the International Committee of Medical Journal Editors (ICMJE) were ensured under our Ethics and Research Institutional Committee (IRB) approval.

This study was presented to and approved by both our institution’s research committee CIMED and the Universidad del Rosario’s ethics committee under number CEI DVO005 2207-CV1670.

Written informed consent was obtained from the patient before realizing this manuscript for both case description and obtained images, approving the publication of this manuscript. Information revealing the subject’s identity was avoided.

## Data availability statement

All data generated or analyzed during this study are included in this article and its supplementary material. Further enquiries can be directed to the corresponding author.

## CRediT authorship contribution statement

**Isabella Van-Londoño:** Writing – review & editing, Writing – original draft, Methodology, Investigation, Formal analysis, Data curation, Conceptualization. **Camilo Ramírez-Giraldo:** Writing – review & editing, Writing – original draft, Methodology, Formal analysis, Data curation. **Julio César Martínez Echeverri:** Writing – original draft, Data curation. **Juan José Villany-Sarmiento:** Writing – original draft, Data curation. **Laura Marcela Fino-Velásquez:** Writing – original draft.

## Declaration of competing interest

The authors declare that they have no known competing financial interests or personal relationships that could have appeared to influence the work reported in this paper.
