# Supplementary material for: Eosinophilic enterocolitis in duodenum, ileum, and colon: A case report
Source: Heliyon. 2024 Mar 2;10(5):e26885. doi: 10.1016/j.heliyon.2024.e26885 (PMC10925976; doi:10.1016/j.heliyon.2024.e26885)
Supplement: Multimedia component 1 [file mmc1.docx]

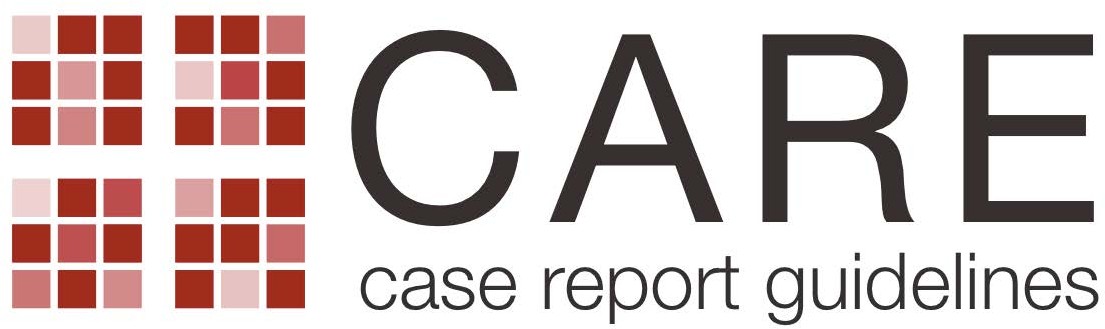
CARE Checklist of information to include when writing a case report
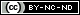


**Topic Item Checklist item description Reported on Line**

**Title 1** The diagnosis or intervention of primary focus followed by the words “case report” 2

**Key Words 2** 2 to 5 key words that identify diagnoses or interventions in this case report, including "case report" 2

**Abstract**

**(no references)**

**3a** Introduction: What is unique about this case and what does it add to the scientific literature? 41-42

**3b** Main symptoms and/or important clinical findings 41-42

**3c** The main diagnoses, therapeutic interventions, and outcomes 41-43

**3d** Conclusion—What is the main “take-away” lesson(s) from this case? 43-44

**Introduction 4** One or two paragraphs summarizing why this case is unique (**may include** reference**s**) 66-88

**Patient Information 5a** De-identified patient specific information 91

**5b** Primary concerns and symptoms of the patient 91-96

**5c** Medical, family, and psycho-social history including relevant genetic information 91-92

**5d** Relevant past interventions with outcomes 95-96

**Clinical Findings**

**Timeline**

**Diagnostic Assessment**

**Therapeutic Intervention**

**Follow-up and Outcomes**

1. Describe significant physical examination (PE) and important clinical findings 94-129
2. Historical and current information from this episode of care organized as a timeline 94-143

**8a** Diagnostic testing (such as PE, laboratory testing, imaging, surveys). 96-132

**8b** Diagnostic challenges (such as access to testing, financial, or cultural) 144-147, 194-216

**8c** Diagnosis (including other diagnoses considered) 99-100, 109, 114, 129,131

**8d** Prognosis (such as staging in oncology) where applicable Non applicable

**9a** Types of therapeutic intervention (such as pharmacologic, surgical, preventive, self-care) 107, 112-13, 117 140-141

**9b** Administration of therapeutic intervention (such as dosage, strength, duration) 107, 117, 140-141

**9c** Changes in therapeutic intervention (with rationale) 116-17, 140-143

**10a** Clinician and patient-assessed outcomes (if available) 140-146

**10b** Important follow-up diagnostic and other test results 144-147

**10c** Intervention adherence and tolerability (How was this assessed?) Non applicable

**10d** Adverse and unanticipated events Non applicable

**Discussion 11a** A scientific discussion of the strengths AND limitations associated with this case report 181-220

**11b** Discussion of the relevant medical literature **with references** 152-192

**11c** The scientific rationale for any conclusions (including assessment of possible causes) 188-92, 194-208

**11d** The primary “take-away” lessons of this case report (without references) in a one paragraph conclusion 210-216

**Patient Perspective 12** The patient should share their perspective in one to two paragraphs on the treatment(s) they received 144-146

**Informed Consent 13** Did the patient give informed consent? Please provide if requested . . . . . . . . . . . . . . . . . . . . . . . . . . . . . . . . . . . . . . **Yes.** ✓ **No**
